# Editorial: Mind-body medicine and its impacts on psychological networks, quality of life, and health, Volume II

**DOI:** 10.3389/fnint.2025.1658381

**Published:** 2025-07-23

**Authors:** Steffen Schulz, Dirk Cysarz, Frauke Musial, Georg Seifert

**Affiliations:** ^1^Charité Competence Center for Traditional and Integrative Medicine (CCCTIM), Charité – Universitätsmedizin Berlin, Corporate Member of Freie Universität Berlin, Humboldt-Universität zu Berlin and Berlin Institute of Health, Berlin, Germany; ^2^Integrated Curriculum for Anthroposophic Medicine, Faculty of Health, Witten/Herdecke University, Witten, Germany; ^3^The National Research Center in Complementary and Alternative Medicine (NAFKAM), Department of Community Medicine, UiT, The Arctic University of Norway, Tromsø, Norway; ^4^Department of Pediatrics, Faculty of Medicine, University of São Paulo, São Paulo, Brazil

**Keywords:** mind-body medicine, health and chronic diseases, emotions, lifestyle and behavior, evidence-based, integrative medicine, network physiology, psychology

This Research Topic is the second volume of the series “*Mind-body medicine and its impacts on psychological networks, quality of life, and health*” (Schulz et al., 2023a,b).

The improvement in living conditions, particularly in industrialized countries, has resulted in a substantial increase in life expectancy over recent decades. Concurrently, the prevalence of chronic diseases is escalating. These include cardiovascular diseases, chronic pain, inflammatory bowel diseases and cancer. The manifestation and chronification of disease is precipitated by a combination of detrimental lifestyle factors, including but not limited to: cumulative stress, inadequate exercise, and suboptimal nutrition. These factors, when compounded by an imbalance between exertion and recovery, can have a profound impact on health outcomes. There has been a marked increase in the level of awareness regarding the interconnection between the human mind, emotional state, lifestyle choices and physical health. The understanding of body and mind interaction is increasing, and this is supported and confirmed by evidence demonstrating a high level of clinical relevance. The development of mind-body medicine (MBM) is intimately associated with this field of research.

MBM is a holistic approach that integrates psychological and physical methodologies with the objective of enhancing health and wellbeing. This field encompasses practices such as meditation, yoga, and biofeedback and many more, which aim to enhance the mind's capacity to affect bodily functions and symptoms. Recent research has demonstrated that MBM can significantly influence neural networks, leading to improved mental health outcomes. The efficacy of these practices in reducing stress, anxiety and depression has been demonstrated, thereby enhancing overall quality of life. Moreover, MBM interventions have been demonstrated to exert a favorable influence on physical health, as evidenced by a reduction in blood pressure, enhancement of immune function, and alleviation of chronic pain. MBM offers a variety of benefits for mental and physical health. Some of the main ones are stress reduction, improved mental health, increased quality of life, physical health benefits, promoting self-awareness and takes a holistic approach to health promotion, addressing both the mind and body for more comprehensive healing.

The MBM approach is predicated on the premise that there is an interplay between the body, mind, emotions and behavior. This interplay extends to the regulation of vegetative physiological signaling pathways. The objective of MBM is to utilize scientifically-based knowledge on the interactions between the body, mind and emotions to promote salutogenetically-based resources. In this theoretical framework, the body, mind and emotions are regarded as operating in unison, with illnesses being conceptualized as inducing a state of imbalance within complex bio-psychological systems. A fundamental tenet of MBM emphasizes the necessity for lifestyle modifications. The psychological aspects of MBM have been demonstrated to exert an influence on lifestyle factors, and thus exert an indirect effect on a wide range of areas of the mind-body connection. A range of techniques are employed within MBM, including but not limited to: mindfulness-based stress reduction, meditation, active movement, relaxation, mindfulness, stress management, relaxation and habits of perception and evaluation. These interventions are designed to target multifaceted bio-psychological and social systems, encompassing diverse domains of lifestyle, with the objective of enhancing self-efficacy and promoting health. The therapeutic approaches employed by MBM comprise a variety of methods that frequently facilitate the provision of cost-effective preventive or therapeutic options. As demonstrated by the existence of relevant evidence-based data, this field has the potential for significant scientific and clinical growth in relation to lifestyle change and the prevention of some of the most significant health issues. MBM interventions are employed within both conventional medicine and complementary medicine, with their application arising from emotional, mental, social, spiritual and behavioral factors. These treatments are also often combined to influence health. The analysis of these signaling pathways has revealed three distinct mechanisms: structural, dynamic and regulatory. The information transfer in both healthy and diseased states provides insights into the physiological structures and functions of the whole integrated system, with its different types of interaction (Schulz et al., 2018, 2019).

The second volume of the series “*Mind-body medicine and its impacts on psychological networks, quality of life, and health*” focuses on the evidence-based investigations on physical exercise, integrative strategies for self-care, applications from traditional Chinese medicine, hypnotherapy, Ayurveda, relaxation, meditation methods, yoga practice, qigong, tai chi, biofeedback interventions, implementation of digital health tools, evaluation of behavioral change techniques, mindful stress relief, cognitive restructuring, autogenic training, and health-impacting social support. The present Research Topic has amassed a wide array of research content, encompassing multidisciplinary contributions within the domain of MBM. This includes comprehensive data analysis and clinical practice applications. The present volume II includes 40 articles and comprises 26 original research articles, one methods article, one hypothesis and theory article, two perspective articles, one mini review and 9 systematic reviews. Participating journals were Frontiers in Integrative Neuroscience, Frontiers in Behavioral Neuroscience, Frontiers in Medicine, Frontiers in Network Physiology, Frontiers in Psychiatry, and Frontiers in Public Health.

The content of the second volume of the series “*Mind-body medicine and its impacts on psychological networks, quality of life, and health*,” can be clustered within four main categories.

Interventions for mental health and wellbeing: This category comprises studies that investigated diverse interventions or factors that significantly influence mental health, emotional states, stress, and overall wellbeing, frequently incorporating a mind-body component or emphasizing resilience. The following key issues were addressed by this category: physical exercise and emotional wellbeing; self-reflection and stress reduction; integrated physical-mental therapies; mindfulness and wellbeing in adolescents; mindful movement for mental health; Yoga for stress and emotional wellbeing; mind-body exercises and quality of life; creative arts and emotional regulation; patient consciousness in therapy; health and wellness coaching; and group meditation for public health.

Specific conditions and symptoms: depression, anxiety, stress, and fatigue: This category encompasses studies that addressed specific mental health disorders or key symptoms, frequently exploring their prevalence, interconnections, risk factors, or targeted interventions. The following key issues were addressed by this category: The following studies have been conducted on the subject of cancer-related fatigue and stress: eurythmy therapy, activity-based stress release, psychosomatic health in students, depression in cancer patients, automated stress detection, sex differences in diabetes patients' symptom networks, anxiety, depression, sleep in university students, BMI and depressive symptoms, mental health literacy and psychological status, Traditional Chinese Medicine (TCM) and depression, adolescent suicide attempters, exercise for COVID-19 patients' mental health, and mind-body therapies for adolescent depression.

The connection between physical health, pain and quality of life and mental health: This category includes studies that focused on physical wellbeing, pain management, and general quality of life, often exploring their connections to psychological factors, social support, or mind-body interventions. The following key issues were addressed by this category: sleep duration and chronic musculoskeletal pain, Tai Chi vs. water aerobics and brain connectivity, social support and quality of life in cancer patients, health-related quality of life assessment in alopecia areata, placebo effects and appetite/satiety, physiological markers for social and physical pain, social alienation and emotional intelligence, placebo in mindfulness-based interventions for pain, exercise enjoyment in overweight/obese individuals, breakfast consumption and handgrip strength, Qigong for COVID-19 rehabilitation, mind-body exercise for stroke patients, outdoor interventions for myopia, and inter-organ regulation of affective states.

The connection between physical and mental health in an educational context: This category specifically includes studies in which the study population is comprised of students, often addressing their unique challenges, academic expectations, or specific interventions relevant to their developmental stage. The following key issues were addressed by this category: doctoral students' expectations, physical exercise and emotional states in university students, mindful movement for kinesiology and sport students, psychosomatic health in TCM university students, Yoga for university students' wellbeing, mental health symptoms and health-promoting lifestyles in Chinese university students, and Qi states and depression in college students.

## Interventions for mental health and wellbeing

Qian et al. investigated the psychophysiological effects of NaiKan Therapy, a self-reflection method, on salivary oxytocin and cortisol release in 60 participants over a period of 5 days. A significant increase in oxytocin levels and a decrease in cortisol levels were observed post-therapy, suggesting that NaiKan Therapy enhances social bonding and reduces stress reactivity. These findings offer novel insights into the neuroendocrine mechanisms of this introspective practice and its potential for improving mental wellbeing. Singh et al. introduced a Budo group therapy for psychiatric in- and outpatients, incorporating martial arts, mindfulness, and breath work to enhance physical and mental health. Over a 14-month period, 215 individuals participated, demonstrating good retention and high self-reported satisfaction, motivation, and improved physical and psychological wellbeing. The study concludes that Budo group therapy is a feasible, well-accepted, and promising transdiagnostic treatment approach that combines physical activation with resilience enhancement for a broad spectrum of mental health needs. Ecker et al. investigated the immediate effects of a single mindfulness intervention on healthy adolescents aged 12–19, using both subjective and physiological measurements. Contrary to the prevailing expectations, no significant immediate improvements in wellbeing, state mindfulness, heart rate, or heart rate variability were observed in comparison to an active control group. However, the findings indicated that a generally mindful attitude (trait mindfulness) was positively correlated with better subjective wellbeing and reduced mental burden in this age group, suggesting its potential as a resilience factor. Spaccapanico Proietti et al. evaluated the short-term impact of an 8-week mindful movement programme (Movimento Biologico) on 38 Kinesiology and Sport Sciences students. The programme led to a marked enhancement in the positive mental health of the participants, as well as in several interoceptive awareness subscales, including emotional awareness. Furthermore, the autonomy subscale of psychological wellbeing demonstrated a significant enhancement, indicating an improvement in body sensation recognition, emotional-physical links, and self-confidence. Castellote-Caballero et al. analyzed, in a randomized controlled trial, the efficacy of a 12-week yoga intervention on stress, emotional wellbeing, and anxiety in 129 university students. The experimental group demonstrated significant enhancements in perceived stress, emotional wellbeing, and both state and trait anxiety, in comparison to the control group. Their finding indicated that yoga can play a substantial role in the reduction of stress and anxiety, and the enhancement of emotional wellbeing in university students. Yang et al. investigated the impact of mind-body exercises on the quality of life in 1,087 older adults, exploring the chain mediating effects of perceived social support and psychological resilience. They found a significant and positive correlation between quality of life and mind-body exercises. Their findings indicated that perceived social support and psychological resilience exhibited a chain mediating effect, signifying that mind-body exercises enhance quality of life by fortifying these factors. Barnett and Vasiu investigated, in a hypothesis and theory paper, the neural mechanisms of creative arts' therapeutic effects on mental and physical health, with a specific focus on the medial prefrontal cortex (mPFC) and amygdala. Preliminary findings indicate that both active and passive engagement with creative arts consistently activate neural circuits involved in adaptive emotional regulation. The study concludes that creative arts have the potential to function as a complementary therapeutic strategy by engaging shared neural mechanisms with emotional regulation, thereby enhancing understanding of their role in mental health. Shirbache et al. introduced “Ultra-Overt Therapy” (UOT), a novel medical approach that emphasizes patient consciousness and awareness of medication's physiological processes to improve drug efficacy. Their non-systematic review explored evidence from the mind-body relationship, placebo response, neuroscience, and complementary medicine, suggesting theoretical promise for UOT. It is recommended that future research be conducted to ensure a comprehensive understanding of the global impact of this method on medical treatment and patient care. Wolever and Weinand presented the Health and Wellness Coaching (HWC) as an evidence-based approach to address chronic disease, integrating mind-body science and autonomy-promoting lifestyle interventions. The Vanderbilt Health Coaching Program's process is detailed, highlighting the integration of mindfulness, the Wheel of Health, and guided visualization with motivational interviewing. Additionally, structural tools like the Vanderbilt Health Coaching Funnel and IVA Funnel are introduced to foster sustainable behavior change and clarify true coaching strategies. Schneider et al. underscored, in a perspective paper, the pressing need for novel public health strategies to avert collective stress, violence, and war, examining the role of evidence-based meditation from Ayurveda and Yoga in this context. The programme under scrutiny is that of Transcendental Meditation, which has been demonstrated to be effective in reducing collective stress and conflict while improving quality of life, as evidenced by empirical studies across cultures. The mechanisms by which group meditation mitigates collective violence are explored through the lenses of public health models, neuroscience, and quantum physics principles. This suggests that group meditation has the potential to enhance societal wellbeing and peace.

## Specific conditions and symptoms: depression, anxiety, stress, and fatigue

Timm et al.b investigated in a prospective observational study the online implementation of eurythmy therapy (ERYT), a mindful-movement therapy from anthroposophic medicine, for cancer-related fatigue (CRF), stress, and mindfulness. Study 1, which utilized a mixed sample, demonstrated enhancements in emotional and physical wellbeing, with a concomitant reduction in stress and an increase in mindfulness. However, the study did not reveal a significant effect on fatigue. Study 2, which focused on subjects diagnosed with cancer, revealed a substantial enhancement in CRF, stress, and mindfulness scores. These findings imply that online ERYT may offer benefits for these indicators in cancer patients and underscore the accessibility advantages of the online format. In another study (Timm et al.a) they appraised the practicability and efficacy of an 8-week online Activity-Based Stress Release (ABSR) programme, a pioneering mindfulness-based intervention (MBI) derived from anthroposophic medicine, on perceived stress and mindfulness. A large-scale observational study with 830 participants was conducted, and the results demonstrated a significant decrease in self-reported stress and a substantial increase in mindfulness scores over the course of the intervention. These benefits were sustained at follow-up. They suggested that this AM-based intervention effectively cultivates mindfulness and is adaptable to an online format, offering a diverse approach within MBIs. Byun et al. evaluated the feasibility of automated stress detection in patients diagnosed with major depressive disorder (MDD), panic disorder (PD), and healthy controls (HCs) using machine-learning algorithms based on heart rate variability (HRV) features. A total of 147 subjects participated in the study, during which HRV data was collected during stress and relaxation tasks. The highest classification accuracies were observed in healthy controls (HCs), however, personalized longitudinal scaling significantly improved accuracies across all groups to over 0.90. They suggested that HRV metrics have the potential to serve as biomarkers of stress; however, the altered autonomic responses observed in psychiatric patients necessitate the development of tailored approaches for stress monitoring. This emphasizes the value of longitudinal scaling for the development of personalized technologies. Wu et al. investigated sex differences in the symptom network structure of depression, anxiety, and self-efficacy among 413 diabetes patients. The prevalence of depression and anxiety was found to be higher in female subjects than in male subjects. The strongest symptom connections exhibited variation by sex; nevertheless, “worry” and “nervousness” were found to be central to both groups. They found a strong negative association between “guilt” and self-efficacy in females, suggesting the need for targeted interventions to promote psychological wellbeing. Jafari et al. investigated, in a cross-sectional study, mental health literacy (MHL) and depression literacy (D-Lit) and their relationship with psychological status and quality of life in 400 Iranian patients with type 2 diabetes mellitus (T2DM). They revealed inadequate levels of MHL and D-Lit, with only 5.8% of respondents answering D-Lit questions correctly. Their findings indicated a robust correlation between low MHL and D-Lit with elevated symptoms of depression, anxiety, and stress, as well as a diminished quality of life. This underscores the imperative for targeted mental health interventions. Tomaszek et al. examined, in a retrospective cohort study, the demographic and clinical profiles of 425 adolescent suicide attempters (aged 11–17 years) who were admitted to an emergency department during the period of the COVID-19 pandemic. The majority of participants were female, aged between 15 and 17, and resided in urban areas. The most prevalent method was self-poisoning, frequently involving antidepressants or paracetamol, followed by self-harm. It is noteworthy that approximately 70% of visits were linked to mental disorders, primarily depressive disorder, thereby underscoring the pandemic's enduring repercussions on youth mental health. Tang et al. assessed, in a systematic review and meta-analysis, the impact of exercise therapy on anxiety and depression in patients with COVD-19. A meta-analysis of six studies involving 461 patients with confirmed cases of the disease found that those who engaged in regular exercise demonstrated significantly reduced anxiety levels, depression levels, PHQ-9 scores, and sleep quality when compared to non-exercising individuals. The research provides evidence that exercise therapy can effectively help patients with confirmed cases of the novel coronavirus (SARS-CoV-2) to experience reduced symptoms of anxiety and depression, and to achieve better sleep quality. Luo S. et al. investigated, in a systematic review and network meta-analysis, the effectiveness of different mind-body therapies (MBTs) in alleviating depression among adolescents. A comprehensive review of the extant literature was conducted, encompassing nine randomized controlled trials with a total of 955 subjects. Their findings indicated that yoga, dance therapy and Tai Chi were more effective in reducing depressive symptoms. Specifically, yoga was identified as the optimal intervention, followed by dance therapy and Tai Chi, demonstrating positive effects on adolescent depression. Moshfeghinia et al. investigated, in a systematic review and meta-analysis, the association between depression and keratoconus (KC), a chronic corneal disease. A comprehensive analysis encompassing 83 KC patients and 3,186 controls yielded a conspicuously elevated depression score in the KC cohort. However, a meta-analysis of four studies comparing depression rates found no increased overall risk of depression among KC patients. Their findings suggest a complex relationship between KC and mental health, warranting further investigation.

## The connection between physical health, pain and quality of life and mental health

Li et al. investigated in a cross-sectional study the relationship between sleep duration and chronic musculoskeletal pain (CMP) in 3,904 US adults using NHANES data. The study revealed a U-shaped association, indicating that both short (<7 h) and long (≥9 h) sleep durations were associated with an increased prevalence of CMP, with 7 h of sleep exhibiting the lowest odds ratio. These findings underscore the importance of maintaining optimal sleep duration for the promotion of musculoskeletal health. Port et al. made a comparison between the functional brain connectivity of older adults who practice Tai Chi and those who practice Water Aerobics in a case-control fMRI study. They demonstrated that practitioners of Tai Chi exhibited stronger connectivity in the Salience Network during periods of rest. Furthermore, increased correlations were observed in brain regions associated with memory, attention, and cognitive control during the performance of the N-Back and Stroop tasks. In contrast, the Water Aerobics group demonstrated enhanced connectivity in areas associated with motor actions and object mirroring during the Stroop task, suggesting the presence of distinct neural mechanisms for these activities. Mandato et al. conducted a longitudinal study with a view to evaluating the impact of medical history, perceived physician-patient communication, and perceived social support on quality of life (QoL) in 98 endometrial cancer (EC) patients during their 1^st^ year following surgery. They found that elevated perceived social support was associated with enhanced emotional wellbeing (EWB) at 1 month and 1 year following surgery. Furthermore, support from a significant other was found to be associated with improved physical functioning, reduced pain, and diminished fatigue at 1 year. The study concludes that multifaceted social support is a pivotal factor in bolstering psychological wellbeing and enhancing overall QoL for EC patients, emphasizing its importance in comprehensive care. Muhetaer et al. utilized a network approach to explore the inter-relationship between depression and health-related quality of life (HRQOL) dimensions in 1,735 Chinese cancer patients. The prevalence of depression, a central symptom that manifested alongside symptoms such as nausea/vomiting, pain, and physical function, was found to be nearly two-thirds of patients. The impact of depression on health-related quality of life (HRQOL) is predominantly through its effect on emotional function, pain, physical function and sleeplessness. This underscores the significance of timely treatment for depression in enhancing overall HRQOL. Caldarol et al. conducted a multicentric study with the aim of evaluating the psychometric properties of the Italian Skindex-16AA in patients diagnosed with moderate-to-severe Alopecia Areata (AA). The analysis yielded a two-factor, eight-item structure, designated Skindex-8AA, which exhibited satisfactory psychometric properties, including internal consistency, convergent validity, and test-retest reliability. The Skindex-8AA was proposed as a suitable instrument for the assessment of HRQOL in AA patients. Cui et al. analyzed, in a cross-sectional study, data from 10,686 Chinese adults in order to investigate the relationship between body mass index (BMI) and depressive symptoms. They demonstrated a substantial U-shaped correlation, suggesting that both underweight and obesity elevated the risk of depression. This association was particularly pronounced in younger, highly educated, single, and employed subgroups, suggesting that maintaining a normal body weight is a crucial strategy for preventing depression and promoting overall physical and mental health. Lanz et al. tested the hypothesis, in a double-blind study, if placebo-induced changes in appetite and satiety could influence attentional bias toward food cues in a group of 63 healthy participants. Participants received a placebo capsule with specific expectancy manipulations for enhanced appetite or satiety, followed by a visual probe task to measure attentional bias. The results demonstrated that placebo-induced satiety effectively hindered attention allocation toward food in healthy women, as evidenced by significantly elevated reaction times for food cues in comparison to non-food cues. This process may be mediated by a reduction in hunger and food craving. Lopes et al. examined the role of placebo effects and expectations in mindfulness-based interventions (MBIs) for pain, given their established effectiveness but unclear mechanisms. A total of 19 studies were included in the review, but only a small number of these specifically focused on MBI-related placebo effects. However, the studies indicated a clear role for placebo and expectations in MBI outcomes for both acute and chronic pain. They emphasized the necessity for these factors to be considered routinely in future experimental designs and further research to be conducted in order to achieve a comprehensive understanding of the connection between MBIs, placebo/expectations, and pain relief. Jang et al. (2024) compared multimodal physiological responses to social and physical pain in a group of 73 healthy participants. The study found that social pain induced increased heart rate (HR) and skin conductance, and decreased blood volume pulse, pulse transit time, respiration rate (RR), and finger temperature (FT). The physical pain induced an increase in heart rate variability and skin conductance, as well as a decrease in blood volume pulse and pulse transit time. However, no change was observed in FT. The presence of these distinct patterns indicates that HR, HRV indices, RR, and FT can serve as markers to differentiate physiological responses to social and physical pain stimuli. Diao et al. evaluated the levels of social alienation experienced by patients undergoing peritoneal dialysis. In addition, their investigation sought to ascertain the mediating factors that contribute to this phenomenon, namely personal mastery and perceived social support. The results indicated a mean social alienation score of 42.01 ± 3.15. Elevated emotional intelligence levels were found to be significantly correlated with reduced social alienation. The mediation model demonstrated that personal mastery and perceived social support fully mediated this effect, highlighting their importance for interventions. Luo Y. et al. evaluated and compared, in a meta-analysis, the effects of High-Intensity Interval Training (HIIT) and Moderate-Intensity Continuous Training (MICT) on enjoyment and affective responses in overweight or obese individuals. Including 16 articles with 537 participants, the study analyzed enjoyment using the Physical Activity Enjoyment Scale and affective responses via the Feeling Scale and Felt Arousal Scale. The review concluded that HIIT generally yields a better pleasure response than MICT in this population, though no significant difference in emotional response was found. Ren et al. elucidated, in a systematic review and meta-analysis, the conflicting evidence regarding the association between the frequency of breakfast consumption and handgrip strength, as well as standing long jump. The meta-analysis, which incorporated a total of six studies on grip strength and three on standing long jump, revealed a significant positive association between regular breakfast consumption and higher handgrip strength levels in women, though not in men. No significant differences were observed in standing long jump performance based on breakfast consumption frequency for either sex. Antonelli and Donelli investigated, in a systematic review, Qigong's potential as integrative support for COVID-19 and Long-COVID-19 rehabilitation. The review of pertinent clinical studies revealed beneficial effects of Qigong, an ancient Chinese practice combining movements, breathing, and meditation, on persistent respiratory issues, dizziness, sleep disturbances, and health-related quality of life. Further investigation is crucial to quantify and standardize Qigong's contribution, aiming to integrate this accessible practice into public health strategies and comprehensive treatment regimens. Dong et al. systematically evaluated, in a systematic review and meta-analysis, the effects of mind-body exercise on physical ability, mental health, and quality of life in stroke patients. A total of 33 randomized controlled trials with 1985 participants were included, revealing significant improvements in balance, upper and lower limb motor ability, overall exercise capacity, depression, and quality of life. However, the impact of mind-body exercise on walking ability was not found to be statistically significant. Notably, Qigong (Baduanjin) with specific intervention parameters was found to be particularly effective in improving balance and quality of life. Mei et al. evaluated, in a systematic review, and meta-analysis the overall efficacy of outdoor interventions for myopia in children and adolescents. Analyzing seven randomized controlled trials with 9,437 subjects, the meta-analysis demonstrated significant improvements in spherical equivalent refraction, axial length, and reduced myopia incidence following outdoor interventions. The study concludes that outdoor interventions effectively prevent and control myopia with low risk and high therapeutic benefits, making them a preferred or adjuvant approach to medication. Arinel and Abdelaal synthesized, in a mini-review, empirical finding on the rapidly developing field of inter-organ regulation of affective and internal states, focusing on the bidirectional communication between the brain, gut, and heart. These conserved mechanisms are crucial for aligning reward states with physiological needs, thereby optimizing survival behaviors such as resource acquisition and adaptation. The review under discussion emphasizes the significance of comprehending the mechanisms and circuits of both gut- and heart-mediated reward processes for the purpose of investigating unconscious and conscious reinforcement, affective disorders, and mind-body interventions.

## The connection between physical and mental health in an educational context

Stock-Schröer and Lange conducted a cross-sectional survey of doctoral students in medicine and health sciences in Germany online to ascertain their expectations for graduate school, with a particular focus on complementary and integrative medicine (CIM). The participants expressed a primary desire for individual personal support, networking opportunities, and mutual support. Medical students placed a higher value on scientific guidance, while non-medical students indicated a preference for personality development and networking. Doctoral students with CIM topics also expressed a desire for improved final grades, with these results being of crucial importance for curriculum development. Cao and Luo explored in a longitudinal study the causal link between physical exercise and emotional states in 1,215 university students, mediated by Sense of Coherence (SOC). Results showed that SOC significantly predicted positive affect (PA), and PA, in turn, predicted physical exercise. Physical exercise also indirectly influenced PA through SOC, underscoring SOC's vital role in promoting emotional wellbeing and the reciprocal relationship between physical activity and positive emotions. Yi et al. investigated the psychosomatic health of 665 TCM university students, revealing high prevalence of depression (41.65%) and anxiety (36.69%), alongside common somatic symptoms. Network analysis identified “worrying too much,” “uncontrollable worries,” and “weakness” as central symptoms within the comorbid network. The identification of “little interest or pleasure,” “feeling down,” “dyssomnia,” and “sighing” as bridging symptoms suggests that these are crucial targets for intervention to prevent mutual symptom transmission. Sun et al. employed network analysis to identify core and bridge symptoms within comorbid anxiety, depression, and sleep problems, and to explore their interconnections with health-promoting lifestyles (HPLs) among 3,896 Chinese university students. The study identified “low energy,” “daytime dysfunction,” and “trouble relaxing” as core symptoms, while “physical activity,” “spiritual growth,” and “stress management” emerged as key health-promoting behaviors. Their findings indicated that the targeting of these core/bridge symptoms and the promotion of specific HPLs can significantly enhance the mental health of university students. Xinzhu and Yuanchun ascertained the correlations between the following three variables: Qi stagnation, Qi deficiency, and depression levels. The study population comprised 403 college students. Their findings indicated the presence of mild depressive symptoms, low levels of Qi stagnation and deficiency, and a strong positive correlation between the two Qi states. They found a moderate and positive correlation between both Qi stagnation and Qi deficiency with depression, thereby providing support for the traditional Chinese medicine theory and suggesting that physical therapy may be an effective intervention for alleviating symptoms.

In summary, the thematic content and focus of the series was primarily on the following areas: the mind-body connection, mind-body networks and mindfulness-based approaches; mental health and wellbeing (in a general sense and with regard to symptoms); physical health and physiology; social aspects and support; methodology and diagnostics; neurobiological and psychophysiological mechanisms; and specific populations and contexts. The clinical questions focused on specific research questions or hypotheses, such as the effectiveness of interventions, prevalence, associations and correlations, mechanisms and biomarkers, development and validation of measurement tools, and feasibility and acceptability. The diseases/conditions under scrutiny encompassed a wide range of ailments, including, but not limited to, mental health and mood disorders, such as depression and anxiety, stress and panic disorders, suicide attempts, social alienation, affective disorders, and sleep problems. In addition to physical illnesses, the following conditions have been observed: chronic musculoskeletal pain, pain (general/specific), endometrial cancer, cancer (general/Chinese cancer patients), alopecia areata, diabetes (type 2), long-term effects of severe acute respiratory syndrome (SARS-CoV-2) infection, stroke, myopia, overweight/obesity and chronic diseases (general). The studies under review in this series make reference to a number of interventions, including but not limited to mind-body therapies such as Yoga, Tai Chi, Qigong, mindfulness-based interventions, eurythmy therapy, Budo group therapy, NaiKan therapy (self-reflection), dance therapy, transcendental meditation, creative arts, health and wellness coaching, and ultra-overt therapy; physical activity and exercise such as general physical exercise, water aerobics, high-intensity interval training, continuous moderate-intensity exercise, exercise therapy, and outdoor interventions. The following factors have been identified as playing a role in the provision of psychosocial support and lifestyle interventions: social support, maintaining normal body weight, maintaining optimal sleep duration, health-promoting lifestyles, targeted psychological interventions, physiotherapy, placebo effects and expectations (as intervention factors); and technological and personalized approaches, such as automated stress detection and personalized technologies (based on HRV).

The second volume of the series “*Mind-body medicine and its impacts on psychological networks, quality of life, and health*,” primarily focused on the mind-body connection and mindfulness-based approaches, examining mental and physical health, social aspects, and related mechanisms. Clinical inquiries addressed intervention effectiveness, prevalence, and underlying mechanisms. A wide array of conditions were investigated, including mental health disorders (e.g., depression, anxiety) and various physical ailments (e.g., chronic pain, cancer, diabetes, SARS-CoV-2 long-term effects). Interventions reviewed encompassed diverse mind-body therapies (e.g., Yoga, Tai Chi, mindfulness), physical activity (e.g., exercise, HIIT), psychosocial support (e.g., social support, lifestyle changes), and technological approaches (e.g., automated stress detection).

## References

[B1] Jang E. H. Eum Y. J. Yoon D. Sohn J. H. Byun S. (2024). Comparing multimodal physiological responses to social and physical pain in healthy participants. Front. Public Health 12:1387056. 10.3389/fpubh.2024.1387056 38638471 PMC11025361

[B2] Schulz S. Bolz M. Bar K. J. Voss A. (2018). Quantification of the central cardiovascular network applying the normalized short-time partial directed coherence approach in healthy subjects. Methods Inf. Med. 57, 129–134. 10.3414/ME17-02-0002 29719920

[B3] Schulz S. Cysarz D. Seifert G. (2023a). Editorial: Mind-body medicine and its impacts on psychological networks, quality of life, and health. Front. Integr. Neurosci. 17:1188638. 10.3389/fnint.2023.1188638 37082088 PMC10111009

[B4] Schulz S. Haueisen J. Bar K. J. Voss A. (2019). Altered causal coupling pathways within the central-autonomic-network in patients suffering from schizophrenia. Entropy 21:e21080733. 10.3390/e21080733 33267447 PMC7515262

[B5] Schulz S. Seifert G. Cysarz D. (2023b). Mind-Body Medicine and Its Impacts on Psychological Networks, Quality of Life, and Health. Lausanne: Frontiers Media SA. 10.3389/978-2-83252-200-4 PMC10111009 37082088

